# The application of complex network theory for resilience improvement of knowledge-intensive supply chains

**DOI:** 10.1007/s12063-023-00365-0

**Published:** 2023-04-10

**Authors:** Jiakuan Chen, Haoyu Wen

**Affiliations:** School of Economics and Management, Xidian University, Xi’an-Shaanxi, China

**Keywords:** Supply chain resilience, Knowledge-intensive supply chain, Complex network theory, Directed weighted network, Resilience improvement paths

## Abstract

With frequent political conflicts and public health emergencies, global supply chains are constantly under risk interference, significantly reducing supply chain resilience (SCR), especially for the knowledge-intensive supply chains (KISCs). To assess and improve the resilience of KISC, this paper uses complex network theory to construct a directed weighted network model suitable for KISC and expresses the SCR as a comprehensive capability that can resist risk and recover from it. Using quantitative indicators plus qualitative assessment to quantify the resilience index and identify the network key nodes. Two resilience improvement paths are proposed for KISCs, improving firms’ development capacity and industrial backup. In the case study, the resilience of the integrated circuit (IC) supply chain is assessed and improved according to real data from the global IC industry. The findings show that (i) The resilience assessment based on the directed weighted network aligns with industrial reality. (ii) Improving firms’ development capability and industrial backup can improve SCR. (iii) Effective improvement of resilience requires targeting key nodes in the supply chain network (SCN). Moreover, the degree of firms’ development capability improvement and industrial backup intensity should be within a specific range.

## Introduction

In recent years, the frequency and intensity of supply chain interference by internal and external risks such as natural disasters and human factors have increased. This effect is more significant for knowledge-intensive supply chains (KISCs), which take knowledge, technology, experience, and information as their core production factors.

The 2011 earthquake in Japan shut down some automotive raw material suppliers. Since the parts produced by these suppliers have core technologies and monopoly patents, the disruption of this production process significantly affected the global automotive supply chain. It shows that when critical participants in the supply chain are affected, it is likely to cause the avalanche phenomenon, which vividly describes the concept of the key nodes in the supply chain (Craighead et al. 2007). The fierce trade war between the US and China in 2019 led many Chinese chemical raw material manufacturers to relocate their factories to other countries in Southeast Asia as the US restricted the number of goods imported from China. This caused losses to Chinese firms and affected the global chemical raw material supply chain. In 2020, COVID-19 exposed the supply chain to many problems (Hald and Coslugeanu 2022), especially leading to the closure of integrated circuit (IC) design, manufacturing, and packaging and testing firms. For example, Malaysia, which occupies 13% of the global packaging and testing market, closed its firms three times, and TSMC’s wafer production capacity was insufficient. These sharply reduced chip production. Moreover, as of 2023, the IC supply chain has yet to recover. Digital technology supply chains, such as the software supply chain and metaverse, are typically KISC today. Due to the global nature of the various technologies and proprietary copyrights in this type of supply chain, the impact can spread quickly and be very costly if something goes wrong at one point. For example, in 2022, in the gaming software supply chain, Blizzard terminated the renewal of its gaming rights with China’s NetEase, resulting in over 100 million Chinese gamers losing their games.

These cases illustrate the current problems faced by KISCs.

1. The network structure of KISC globalization is more vulnerable to political factors and unexpected events (Zhang et al. 2021).
2. KISC participants are often technologically or ecologically bound to each other, making KISCs have key nodes that, when affected, can significantly impact the entire supply chain.
3. The impact of risk on KISCs is significant, so the recoverability of KISCs after risk interference is crucial.

Supply chain resilience (SCR) is the ability of a supply chain to adapt and recover from risk (Hosseini et al. 2019). Properly assessing and improving SCR not only enhances the ability of the supply chain to resist risk but also the ability to recover from it. Therefore, it is increasingly important to assess SCR and investigate the impact of different strategies to improve it, especially in supply chain network (SCN) structures (Kamalahmadi and Parast 2016).

There are two research directions currently for resilience based on SCN. The first sees the need to simulate the real supply network, emphasizing the product flow during operation. Such studies often assessed SCR by constructing a three-level or multi-level network structure that divides supply chain participants into suppliers, manufacturers, and distributors, focusing on the product inventory and transportation processes (Tan et al. 2019; Li et al. 2017). The second is the qualitative assessment method combined with graph theory. Such studies had two focuses. One is to study the structural characteristics of SCN, such as complexity and redundancy (Birkie et al. 2017; Ivanov and Dolgui 2019). The other is to find the factors influencing SCR through expert evaluation methods or explanatory structural models (Jain et al. 2017; Pavlov et al. 2018).

There is no denying that the two types of SCN-based resilience studies are very realistic and practical. However, KISCs have characteristics that distinguish them from other supply chains, leading to the inapplicability of these two research approaches.

(i) In KISC, the production of the product is highly modular, with heterogeneous intermediate products provided by participants at different stages of production. Many supply chain participants have multiple roles, possibly as both suppliers and manufacturers.
(ii) KISC participants often have deep binding relationships and relatively closed ecosystems. Therefore, the critical factors to the resilience of KISCs are the supply chain participants’ relationships. It is meaningless to discuss the structure of the network alone.

The characteristics of KISC bring two implications for current research. On the one hand, the supply chain’s traditional three-level or multi-level network structure cannot accurately describe the location of KISC participants in the network. For example, in the automotive supply chain, firms that produce engines are both suppliers in the production process of cars and manufacturers based on other engine parts. In the digital technology supply chain, many middleware providers are not just suppliers but also developers based on different plug-ins. Thus, it does not make sense to roughly divide the participants in the KISC network into suppliers, manufacturers, and distributors.

On the other hand, the deep binding relationships between KISC participants allow SCR to be not assessed just from the physical structure of the SCN. Furthermore, quantitative variables such as product logistics or inventory cannot explain these relationships. For example, in the IC supply chain, the complexity of the network structure is less important than the degree of technical match between the participants. In the software supply chain or metaverse, product logistics and inventory do not affect its ability to function correctly at all. Moreover, the deep binding relationships resulted in a technological stratification of firms, forming a directional and hierarchical network structure for KISC.

Therefore, there is a need to build an SCN model that aligns with the characteristics of KISCs. The assessment and improvement of resilience should focus on the comprehensive capability of KISCs to cope with risks rather than the network structure characteristics. The following lists the three questions studied in this paper, and we will explain them in detail in Section 4.

1. Build a network model suitable for KISCs
  A directed weighted network is a powerful tool for describing the associative relationships and directions of the network nodes. Therefore, based on complex network theory, this paper establishes a directed weighted network for KISCs. In this network, a class of firms that provide the resources (materials, equipment) required in the product production process is treated as a supply chain participant, denoting a network node. We use the market concentration rate, sales growth rate, and patent number growth rate as quantitative indicators to measure the relationships among supply chain participants, denoting the directed arc weights. This network structure clearly describes the locations and roles of KISC participants and indicates their relationships.
2.  Assess the resilience of KISCs through the established network and identify key nodes
  In this paper, we consider two aspects when assessing SCR: the ability to resist risk when faced with it, i.e., supply chain vulnerability (SCV), and the ability to recover after suffering risk, i.e., supply chain recoverability (SCR*) (Rajesh and Ravi 2015; Vimal et al. 2022). Based on the relationships among SCR, SCV, and SCR* (Birkie et al. 2017; Hosseini et al. 2019; van der Vegt et al. 2015), SCR is negatively correlated with SCV and significantly positively correlated with SCR*. Thus, we represent SCR as a function of SCV and SCR*. Depending on the established network structure, we quantify SCR by assessing the vulnerability index through network cohesion and the recoverability index through the SIR risk propagation model. Pournader et al. (2017) stated that the behavior or changes of specific participants would affect the entire supply chain. Many supply chains increasingly depend on critical firms (Nakatani et al. 2018). For example, the supply chain of Apple mainly depends on Apple itself. Therefore, we also identify the node importance index through the node contraction method to obtain the key nodes in the SCN.
3. Propose and simulate resilience improvement paths for KISCs
  Combining the characteristics of KISCs, we propose two resilience improvement paths. The first is to improve firms’ development capacity, that is, to the KISC participants’ technology and other related capacities. The existence of stable ecological relationships among KISC participants results that a theoretical supplier selection game is not practical (Rajesh and Ravi 2015). So, we start with the supply chain participants’ own capacities. The second is industrial backup, a backup for certain production links in the KISC network. This approach does not create a new SCN but targets critical links in the KISC network, creating substitutability regarding technology and the market.

This paper aims to assess and improve the resilience of KISCs. In our study, we fully consider the characteristics of KISCs, construct a network model suitable for KISCs and assess the resilience using quantitative metrics plus qualitative evaluation. Most importantly, we proposed two resilience improvement paths and verified their effectiveness through numerical simulation.

## Literature review

Currently, SCR has become a research hotspot. This section provides a literature review of relevant concepts and research methods for SCR. In addition, we summarize a table of the network node and edge descriptions from the perspective of SCN.

### Related concepts

Svensson (2000) introduced the concept of SCV, considered it a random interference, and emphasized the need to construct a scientifically rigorous vulnerability theory model. Blackhurst et al. (2018) indicated that SCV is the susceptibility and exposure of the supply chain to disruptive events. Zhang (2021) defined SCV as the impact of risk interference on the effectiveness of the supply chain. Therefore, we understand SCV as the ability of the supply chain to resist risk, a critical dimension in the assessment of SCR. The lower the vulnerability index, the more risk resistant the supply chain is.

On the definition of SCR*. Ho et al. (2015) defined SCR* in three parts, reducing the possibility of disruptions, mitigating the impact of risk interference, and shortening the time to recover the original state. Kamalahmadi and Parast (2017) considered SCR* an adaptive capacity that can restore the supply chain to a stable operating condition by controlling its structure and function. Rajesh and Ravi (2015) stated that SCR* is the ability of the supply system to recover to its original or better state facing risk interference. Therefore, we use recovery time to quantify SCR* and take it as another vital dimension to assess SCR. The time for the supply chain to recover from risk is calculated by the SIR model. A long time means a lower SCR*, the weaker the supply chain’s ability to recover.

The definitions of SCR in the current literature are classified into three categories. The first category defined SCR as an adaptive capacity, which is the ability of a supply chain to withstand and adapt to various risk interference (Hosseini et al. 2019; Pavlov et al. 2018). The second one considered SCR as a recovery mechanism, expressing the ability of a supply chain to recover from internal and external risk interference (Ambulkar et al. 2015; El Baz and Ruel 2021; Ivanov and Sokolov 2019; Raj et al. 2015). The third expressed SCR as a continuity mechanism, the ability to recover from interference and maintain system continuity (Hosseini and Barker 2016; Longo and Ören 2008). Summarizing previous scholars’ definitions of SCR, we found that SCR is usually associated with resisting risk and recovering after suffering it. Therefore, in assessing SCR, we focus on two aspects: the ability to resist when risk comes and the ability to recover after suffering risk.

### Related research

In the SCV assessment, some scholars used directed networks and correlation algorithms to identify SCV factors and key nodes in the supply chain (Mizgier et al. 2013; Nakatani et al. 2018; Ma et al. 2020) developed a quantitative evaluation method through graph theory, mainly for the physical connections of supply chain participants. Wang et al. (2018) used a network attack graph model to simulate the supply chain loss under different attack methods to achieve the SCV assessment. We found that one part of the studies is mainly biased toward identifying impact factors, and another only considers the physical connections of SCN. There is no SCV assessment of directed relational networks, which is an aspect that this paper wants to remedy.

In the study of SCR*. Ivanov and Dolgui (2019) concluded that structural diversity, process flexibility, and node redundancy are critical factors in low deterministic demand-supply chains. Raj et al. (2015) used a survival function to quantify the recovery time of supply chain systems to indicate SCR*. Ivanov et al. (2018) used a control theory approach with optimal recovery planning to demonstrate that scheduling recovery actions are critical in ensuring SCR*. We find that only Raj achieved the quantification of SCR*, and the rest mainly focused on the influencing factors. Simulation is an effective tool to visualize SCR*. So, we simulate the recovery process through the SIR risk propagation model and calculate the recovery time to quantify SCR*.

For the study of SCR, by modeling a real supply chain network, Tan et al. (2019) assessed SCR by measuring the structural redundancy of SCN. Li et al. (2017) represented each supply chain participant as a region and constructed a relationship matrix based on the number of products delivered and the physical distance among regions. SCR is measured by simulating supply chain disruptions through Monte Carlo and calculating the recovery time. Kim et al. (2015) argued that SCR depends on the SCN structure characteristics by studying different network structures, ignoring the correlation between nodes. Pavlov et al. (2018) proposed a new SCR assessment method based on the chain reaction and structural reconstruction approach, focusing only on the number of nodes and edges. Some studies took the various resilience influences summarized as network nodes to build Bayesian networks and achieved more resilient supplier selection and siting strategies (Hosseini and Barker 2016; Lopez and Ishizaka 2019). These studies on SCR focused more on the SCN structure, product logistics, and inventory, ignoring the nodes’ role and the associative relationships among them. Therefore, these network models cannot describe KISCs clearly. What is most important in a KISC network is the deep binding relationships among participants, so it is not very meaningful to study purely in terms of the structural characteristics of the network.

We also summarize the research on resilience from the perspective of SCN, as shown in Table 1, which mainly focuses on the description of network nodes and edges. Firstly, most are based on directed networks and classify the node types into suppliers, manufacturers, and distributors. However, in KISC, participants often have multiple roles, which leads to this network structure not clearly explaining the location and role of KISC participants. Secondly, some use firms as nodes, clearly describing the location of participants in the network. Still, the description of the weight or meaning of the edges does not match the characteristics of KISCs. To compensate for this part of the gap, in the next section, we construct a directed weighted network model that meets the characteristics of KISCs.

**Table 1:** Supply chain network description

**References**	**Graph**	**Nodes**	**Edges**	**Measure**
(Wang and Ip 2009)	Undirected graph	Demand and supply	Delivery lines	The weighted sum of node resilience
(Adenso-Diaz et al. 2018)	Directed graph	Suppliers, plants, wholesalers and customers	Product flows	Network reliability
(Ma et al. 2014)	Undirected graph	Manufacturers, suppliers, retailers and customers	Trading relationships	Node centrality
(Xu et al. 2014)	Directed graph	Firms	Demand-supply relations	Customer satisfaction
(Kim et al. 2015)	Directed graph	Physical locations	Transportation links	The ratio of redundant nodes to total nodes
(Levalle and Nof 2015)	Directed graph	Firms	Material flows	The total cost of flow and total quality of service
(Han and Shin 2016)	Undirected graph	Risk factors	Supply chain relations	Average probability of disruption per risk
(Li et al. 2017)	Directed graph	Suppliers, manufacturers, distribution centers and retailers	Materials or product deliveries	Recovery time
(Nakatani et al. 2018)	Directed graph	Materials	Material-to product links	Market concentration
(Tan et al. 2019)	Directed graph	Materials and factory	Relationship between materials and products	Network fabric redundancy

## Method

**Fig. 1:**
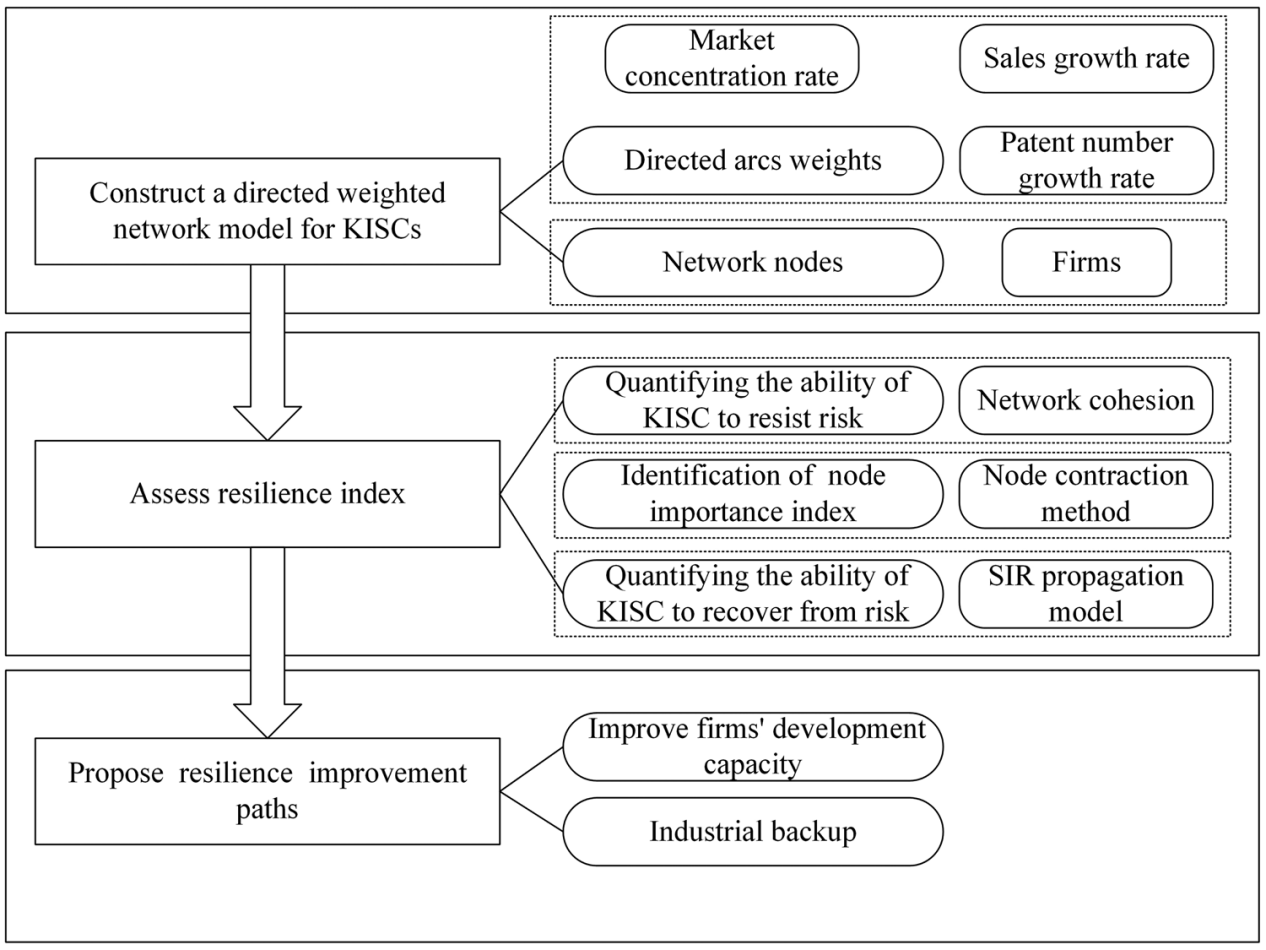
Method flow chart

Firstly, in Section 4.1, we develop a directed weighted network model suitable for KISCs. Secondly, in Section 4.2, we synthesize previous research on SCR and represent it as a comprehensive capability, including the ability of the supply chain to resist risk and recover from it. Moreover, the assessment of resilience is realized by calculating two ability indexes. Finally, by analyzing the characteristics of KISCs, we propose two resilience improvement paths in Section 4.3, including improving firms’ development capacity and industrial backup. The flow chart of the research method is shown in Fig. 1.

### Construction of the directed weighted network

#### Knowledge-intensive supply chain network and its graphical description

Complex network theory is a unique perspective and approach for studying complex systems that focus on the topology of the associative relationships of individuals. It is the basis for understanding the nature of complex systems. Applying complex network theory, we can describe networks in the real world and explain the associative relationships among entities, such as the transportation network of the supply chain and the relationship between people (Ma et al. 2020). Today’s KISCs have highly modular product production and deep binding relationships among participants, making their network structures meet the definition of complex networks.

Considering the difference between KISCs and non-KISCs, we construct a directed weighted network based on the product production process and the required resources. In modeling this, a class of firms that provide the resources (materials, equipment) required in the product production process is treated as a network node. The linkages between the firms are used as directed arcs. The following two sections will explain network nodes and directed arcs in detail. Such a network structure clearly describes the location and role of KISC participants and shows their relationships.

According to complex network theory, the directed weighted network is represented as $\text{G}=\left(\text{V, E, W}\right)$, where$\text{V}=\left\{{\text{v}}_{1},{\text{v}}_{2},{\text{v}}_{3}\dots {\text{v}}_{\text{N}}\right\}$ is the set of nodes,${\text{v}}_{\text{i}}$ represents the $\text{i}$th node in the network, and $\text{N}$ denotes the total number of nodes.$\text{E}=\left\{{\text{e}}_{1},{\text{e}}_{2},{\text{e}}_{3}\dots {\text{e}}_{\text{M}}\right\}$ is the set of directed arcs between nodes,${\text{e}}_{\text{k}}$ denotes the $\text{k}\text{t}\text{h}$ associative relationships between nodes,$\text{M}$is the total number of directed arcs.$\text{W}=\left\{{{\upomega }}_{\text{e}1},{{\upomega }}_{\text{e}2},{{\upomega }}_{\text{e}3}\dots {{\upomega }}_{\text{e}\text{M}}\right\}$ indicates the magnitude of the associative relationships between the nodes, ${{\upomega }}_{\text{e}\text{k}}$ representation is shown in Eq. (1).

1 $${\mathrm\omega}_e=\left\{\begin{array}{lr}\inf&\mathrm{There}\;\mathrm{is}\;\mathrm{no}\;\mathrm{direct}\;\mathrm{relationship}\;\mathrm{between}\;{\mathrm v}_{\mathrm i}\;\mathrm{and}\;{\mathrm v}_{\mathrm j}\\0&{\mathrm v}_{\mathrm i\;}\mathrm{relationship}\;\mathrm{with}\;\mathrm{itself}\\{\mathrm\omega}_{\mathrm{ij}}&\mathrm{There}\;\mathrm{is}\;\mathrm a\;\mathrm{direct}\;\mathrm{relationship}\;\mathrm{between}\;{\mathrm v}_{\mathrm i}\;\mathrm{and}\;{\mathrm v}_{\mathrm j}\end{array}\right.$$

#### Description of network nodes

In modeling SCN, previous studies (Klibi and Martel 2012; Ma et al. 2020; Pournader et al. 2017) were mainly based on the triple network structure, which broadly divided the network nodes into three categories, suppliers, manufacturers, and distributors. However, this way cannot accurately portray the location or role of KISC participants. Nakatani et al. (2018) used life-cycle inventory analysis to express network nodes as resources required in the supply chain and established a reachability matrix between various resources through a life-cycle inventory database. Nakatani’s model can describe the highly modular nature of product production in KISC. Therefore, we adopt Nakatani’s idea of network node description.

We do not classify KISC participants. Based on specifying the resources required in the product production process, a class of firms providing the needed resources is taken as a network node.

#### Description of directed arcs

There are two critical issues about directed arcs in supply chain-directed weighted networks. One is the way of taking values, and the other is the meaning of weights.

In taking values, some scholars mainly considered the physical distance and transportation cost among network nodes (Elluru et al. 2019; Guo et al. 2019). The Global Supply Chain Pressure Index proposed by the New York Fed on January 4, 2022, also mainly considered transportation costs and purchasing managers’ index. However, for KISCs, such as the IC supply chain, the closeness of their technology and market ecology is more important than the cost of transportation. Digital technology supply chains, such as software or metaverse, are not influenced by logistics or product inventory in the real world.

Therefore, we focus more on technology and market ecology when taking values for the directed arcs in the KISC network. We consider the past, present, and future of such firms represented by the nodes and set the directed arc weight ${\upomega }$ as a function of several influencing indicators, including market concentration rate (MCR), sales growth rate (SGR), and patent number growth rate (PGR). The weights of the directed arc represent the size of the development capacity of such firms.

MCR represents the past, indicating the market share of the top n firms (In this paper, we take the top four firms, $\text{MCR}_{4}$), and is a measure of the degree of concentration of the market structure of these firms. A higher MCR value means the node’s market monopoly is more severe and unfavorable for the next associated node. SGR indicates the current business capacity of firms represented by the node. A higher value of SGR means a higher demand for the product at the downstream node, which has a positive relationship with the next associated node. PGR is the current degree of technological innovation and production efficiency of the class of firms represented by the node. Innovation is the key to the development of KISCs, which determines the advancement of product functions and can fully reflect the development trend of firms in the coming decades. Irfan et al. (2019) also said that IT affects the ability to integrate information and coordinate operations in the supply chain. So, PGR is one of the most critical performance factors of KISCs innovation. A higher PGR value indicates that the higher innovation capability of the node is more favorable for the next associated node.

The above analysis shows the influence of the three indicators on the node associative relationships. $\text{MCR}_{4}$ is inversely related to the associative relationships, while SGR and PGR are positively related. Therefore, the directed arc weight ${\upomega }$ is calculated in Eq. (2). The value of ${\upomega }$ indicates the development capacity of firms represented by the nodes. The higher value of ${\upomega }$, the stronger the development capacity of firms.

2 $$\begin{array}{c}\mathrm\omega=\text{SGR}\times\text{PGR}/{\text{MCR}}_4\end{array}$$

Where SGR = sales of this year / sales of last year, PGR = number of patents this year / number of patents last year, $\text{MCR}_{4}={\sum }_{\text{i}=1}^{4}{\text{m}}_{\text{i}}$, ${\text{m}}_{\text{i}}$ indicates the market share of each firm.

There are two ways of interpreting directed arc weights, including similar and dissimilar weights (Tian et al. 2011). Using similar weights, a higher ${\upomega }$ indicates that the distance between the nodes is smaller and the degree of associative relationships is closer. The degree of associative relationships among nodes is the sum of the directed arc weights, while the distance among nodes is the sum of the inverse of the directed arc weights. Dissimilar weights are the opposite of similar weights. We use similar weights to explain the meaning of the directed arc weights. Therefore, if node $\text{i}$ and node j are connected by two directed arcs with weights $\upomega_{\text{ik}}$, $\upomega_{\text{kj}}$, the degree of association $\text{S}_{\text{ij}}$ and the distance $\text{d}_{\text{ij}}$ of the two nodes are shown in Eqs. (3) and (4).

3 $${\text{S}}_\text{ij}={\mathrm\omega}_{\mathrm{ik}}+{\mathrm\omega}_{\mathrm{kj}}$$

4 $${\text{d}}_\text{ij}=1/{\mathrm\omega}_{\mathrm{ik}}+1/{\mathrm\omega}_{\mathrm{kj}}$$

### Quantification of supply chain resilience

We express the resilience of KISCs as a comprehensive capability, including the ability to resist risk when faced with it and the ability to recover after suffering risk. We quantify these two ability indexes in Sections 4.2.1 and 4.2.3. Considering the characteristics of KISC participants, we make the following two hypotheses based on the constructed network model.

#### Hypothesis 1

The firms represented by the nodes in the established directed weighted network are heterogeneous.

#### Hypothesis 2

The nodes in the SCN must include all links of the product production process.

#### Quantification of the supply chain’s ability to resist risk

Zhang (2021) defined SCV as the impact of risk interference on the effectiveness of supply chains. Wagner and Neshat (2012) said that measuring SCV will help managers and public policymakers understand risk exposure. Therefore, we can appreciate SCV as the ability of a supply chain to resist risk when faced with it and use the vulnerability index as one of two critical dimensions in assessing SCR. The higher the vulnerability index, the more likely the supply chain is to be disrupted by risk. This subsection quantifies the vulnerability index using network cohesion.

Network cohesion was first studied in social behavior networks to explore the closeness of relationships between people. Higher cohesion indicates a stronger relationship and less interference from the external environment. As SCN becomes more complex and diverse, network cohesion is beginning to be applied to assess SCV (Carnovale et al. 2019; Huang et al. 2022). There is a deep binding relationship between KISC participants, a crucial point of differentiation from traditional supply chains centered on logistics and product inventory. Therefore, we measure the ability of KISC to resist risk using network cohesion that focuses more on the relationships between participants. The higher the cohesion, the lower the vulnerability index, which means that KISC will be more capable of resisting risks. The network cohesion is calculated by Eq. (5).

5 $${\partial }^{{\upomega }}\left(\text{G}\right)=\frac{1}{\text{S}\times {\text{L}}^{{\upomega }}}=\frac{1}{{\sum }_{1}^{\text{n}}\frac{{\text{S}}_{\text{i}}}{{\text{N}}_{\text{i}}}\times \frac{\sum {\text{d}}_{\text{i}\text{j}}^{{\upomega }}}{\text{n}\left(\text{n}-1\right)}}$$

where ${\partial }^{{\upomega }}\left(\text{G}\right)\left(0<{\partial }^{{\upomega }}\left(\text{G}\right)<1\right)$ is the network cohesion of the directed weighted network $\text{G}$. $\text{S}$ is the sum of the average node association degree of $\text{G}$. ${\text{S}}_{\text{i}}$ is the association degree of node i with neighboring nodes. ${\text{N}}_{\text{i}}$ is the set of neighbors of node $\text{i}$. ${\text{L}}^{{\upomega }}$is the reconciled average shortest distance of the directed weighted network. ${\text{d}}_{\text{i}\text{j}}^{{\upomega }}$ denotes the weighted shortest distance of nodes $\text{i}$ and $\text{j}$.$\text{n}$ represents the total number of nodes in the network.

In assessing the vulnerability index, we should focus on network nodes and consider the associative relationships among them. Therefore, SCV is divided into node network vulnerability and associative relationship network vulnerability. The relationship between the two network structures is shown in the adjacency matrix in Table 2.

**Table 2:** Node network$\text{G}$ and associative relationship network${G}^{\text{*}}$

network	adjacency matrix
Node network$\text{G}$	$$\begin{array}{ccccc}0& {{\upomega }}_{12}& \text{i}\text{n}\text{f}& \text{i}\text{n}\text{f}& \text{i}\text{n}\text{f}\\ \text{i}\text{n}\text{f}& 0& {{\upomega }}_{23}& {{\upomega }}_{24}& \text{i}\text{n}\text{f}\\ \text{i}\text{n}\text{f}& \text{i}\text{n}\text{f}& 0& \text{i}\text{n}\text{f}& {{\upomega }}_{35}\\ \text{i}\text{n}\text{f}& \text{i}\text{n}\text{f}& \text{i}\text{n}\text{f}& 0& {{\upomega }}_{45}\\ \text{i}\text{n}\text{f}& \text{i}\text{n}\text{f}& \text{i}\text{n}\text{f}& \text{i}\text{n}\text{f}& 0\end{array}$$
associative relationship network${\text{G}}^{\text{*}}$	$$\begin{array}{ccccc}0& {{\upomega }}_{12}\times {{\upomega }}_{23}& \text{i}\text{n}\text{f}& {{\upomega }}_{12}\times {{\upomega }}_{24}& \text{i}\text{n}\text{f}\\ \text{i}\text{n}\text{f}& 0& {{\upomega }}_{23}\times {{\upomega }}_{35}& \text{i}\text{n}\text{f}& \text{i}\text{n}\text{f}\\ \text{i}\text{n}\text{f}& \text{i}\text{n}\text{f}& 0& \text{i}\text{n}\text{f}& \text{i}\text{n}\text{f}\\ \text{i}\text{n}\text{f}& \text{i}\text{n}\text{f}& \text{i}\text{n}\text{f}& 0& {{\upomega }}_{24}\times {{\upomega }}_{45}\\ \text{i}\text{n}\text{f}& \text{i}\text{n}\text{f}& \text{i}\text{n}\text{f}& \text{i}\text{n}\text{f}& 0\end{array}$$

We need to calculate the network cohesion of two networks to quantify SCV. The network cohesion ${\partial }^{{\upomega }}\left(\text{G}\right)$ consists of supply chain network nodes, and ${\partial }^{{\upomega }}\left({\text{G}}^\ast\right)$ consists of associative relationships. The specific calculation Eqs. (6) and (7) are as follows.

6 $${\partial }^{{\upomega }}\left(\text{G}\right)=\frac{1}{\text{S}\left(\text{G}\right)\times {\text{L}}^{{\upomega }}\left(\text{G}\right)}=\frac{1}{\sum _{\text{i}=1}^{\text{N}}\frac{1}{{\text{q}}_{\text{i}}}\times \sum _{\text{j}\in {\text{N}}_{\text{i}}}{{\upomega }}_{\text{i}\text{j}}\times \frac{\sum _{\text{i}\ne \text{j}}{\text{d}}_{\text{i}\text{j}}^{{\upomega }}}{\text{N}\left(\text{N}-1\right)}}$$

7 $$\partial^{\mathrm\omega}\left(\text{G}^\text{*}\right)=\frac1{\text{S}\left(\text{G}^\text{*}\right)\times\text{L}^{\mathrm\omega}\left(\text{G}^\text{*}\right)}=\frac1{\sum_{\text{k}=1}^\text{M}\frac1{{\text{q}}_\text{ek}}\times{\sum_{\text{h}\in{\text{M}}_\text{ek}}^{\mathrm\omega}}_\text{ekeh}\times\frac{\sum_{\text{k}\neq\text{h}}\text{d}_\text{ekeh}^{{}^{\mathrm\omega}}}{\text{M}\left(\text{M}-1\right)}}$$

Where $\text{S}$ is the sum of the average node association degree of the directed weighted network. ${\text{L}}^{{\upomega }}$ is the reconciled average shortest distance of the directed weighted network. $\text{q}$ is the number of neighboring nodes of node $\text{i}$. ${\text{d}}^{{\upomega }}$ is the weighted shortest distance between two nodes. $\text{N}$ is the number of nodes in $\text{G}$. ${\text{N}}_{\text{i}}$ denotes the set of neighbors of node ${\text{v}}_{\text{i}}$ in $\text{G}$. ${{\upomega }}_{\text{i}\text{j}}$ is the weight of directed arcs between two nodes in $\text{G}$. $\text{M}$ is the number of nodes in ${\text{G}}^{\text{*}}$, which is equal to the number of directed arcs in $\text{G}$. ${\text{M}}_{\text{e}\text{k}}$ denotes the set of neighbors of node ${\text{v}}_{\text{e}\text{k}}$ in ${\text{G}}^{\text{*}}$. ${{\upomega }}_{\text{e}\text{k}\text{e}\text{h}}$ is the directed arc weight between two nodes in ${\text{G}}^{\text{*}}$. and its value can be determined by the product of the weights of the corresponding connected edges in $\text{G}$.

The vulnerability index is evaluated based on the nodes themselves and the associative relationships among them, as in Eq. (8). we consider node network$\text{G}$ and associative relationship network ${\text{G}}^{\text{*}}$equally important, so ${\upalpha }={\upbeta }=0.5$.

8 $$\mathrm{SCV}=\frac1{\upalpha \ \partial^{\mathrm\omega}\left(\text{G}\right)+\mathrm\beta \ \partial^{\mathrm\omega}\left(\text{G}^\ast\right)}$$

#### Identification of the node importance index in supply chain network

Participants are in the different production processes in KISCs and have different importance. The recoverability of each node after suffering a risk is also different. An impact on a specific participant may bring more significant indirect losses. Identifying the importance index of nodes in a supply chain network can serve as an essential parameter when assessing SCR* and provide a direction for research on SCR improvement.

In identifying the network node importance, some studies argued that the node’s degree distribution plays a vital role in SCN and roughly assume that each node and arc has the same probability of failure (Kim et al. 2015). It does not apply to real-world KISCs. Because in the KISC networks, the location or connectivity of participants is essential, but what is more important is the relationships among participants.

We use the node contraction method to implement the evaluation of the node importance index. The node contraction method is one of the methods to evaluate the importance of nodes in complex networks (Berberler et al. 2021; Jia-sheng et al. 2011). The application of the node contraction method is mainly for some networks where the node contraction does not affect the system operation process. For example, in the software supply chain, middleware is developed by combining several underlying libraries to realize the functional requirements. Therefore, middleware development belongs to its sub-supply chain in the entire software supply chain. A contraction of the middleware node still allows for a clear representation of the entire supply chain operation process. This situation is in line with the highly modular nature of the production of KISC products.

Assuming that each node normally operates in the constructed directed weighted network, the contraction method can contract a node with other nodes it points to into one node. The weights of the contracted directed arc are the product of the two nodes’ directed arc weights. The network structure before and after contraction is shown in Table 3. Calculating the cohesion of the contracted network to achieve node importance assessment.

**Table 3:** Network structure before and after contraction

Network structure before and after contraction	adjacency matrix
Before contraction (assuming contract node 1)	$$\begin{array}{ccccc}0& {{\upomega }}_{12}& \text{i}\text{n}\text{f}& \text{i}\text{n}\text{f}& \text{i}\text{n}\text{f}\\ \text{i}\text{n}\text{f}& 0& {{\upomega }}_{23}& {{\upomega }}_{24}& \text{i}\text{n}\text{f}\\ \text{i}\text{n}\text{f}& \text{i}\text{n}\text{f}& 0& \text{i}\text{n}\text{f}& {{\upomega }}_{35}\\ \text{i}\text{n}\text{f}& \text{i}\text{n}\text{f}& \text{i}\text{n}\text{f}& 0& {{\upomega }}_{45}\\ \text{i}\text{n}\text{f}& \text{i}\text{n}\text{f}& \text{i}\text{n}\text{f}& \text{i}\text{n}\text{f}& 0\end{array}$$
After contraction	$$\begin{array}{cccc}0& {{\upomega }}_{12}\times {{\upomega }}_{23}& {{\upomega }}_{12}\times {{\upomega }}_{24}& \text{i}\text{n}\text{f}\\ \text{i}\text{n}\text{f}& 0& \text{i}\text{n}\text{f}& {{\upomega }}_{35}\\ \text{i}\text{n}\text{f}& \text{i}\text{n}\text{f}& 0& {{\upomega }}_{45}\\ \text{i}\text{n}\text{f}& \text{i}\text{n}\text{f}& \text{i}\text{n}\text{f}& 0\end{array}$$

In the mathematical model, the nodes themselves and the associative relationships (directed arc) owned by the nodes are considered in the node importance identification. The specific calculation steps are as follows.

(i) Calculating the initial network cohesion ${\partial }^{{\upomega }}\left(\text{G}\right)$ and ${\partial }^{{\upomega }}\left({\text{G}}^\ast\right)$ using Eqs. (6) and (7).
(ii) Contraction of node ${\text{v}}_{\text{i}}$ and calculate the contracted network cohesion ${\partial }^{{\upomega }}\left(\text{G}\left({\text{v}}_{\text{i}}\right)\right)$ and ${\partial }^{{\upomega }}\left({\text{G}}^\ast\left({\text{v}}_{\text{i}}\right)\right)$.
(iii) According to Eqs. (9), (10), and (11), assess the important index of node ${\text{v}}_{\text{i}}$.
(iv) Executing operation (ii) (iii) for each node in the network.

9 $$\begin{array}{c}\mathrm{IMC}\left({\text{v}}_\text{i}\right)={\text{IMC}}_\text{G}\left({\text{v}}_\text{i}\right)+\sum\limits_{\text{k}\in\text{M}_\text{ek}^\text{i}}{\text{IMC}}_{\text{G}^\text{*}}\left({\text{v}}_\text{ek}\right)/{\text{q}}_\text{i}\end{array}$$

10 $${\text{IMC}}_\text{G}\left({\text{v}}_\text{i}\right)=1-\partial^{\mathrm\omega}\left(\text{G}\right)/\partial^{\mathrm\omega}\left(\text{G}\left({\text{v}}_\text{i}\right)\right)$$

11 $${\text{IMC}}_{\text{G}^\ast}\left({\text{v}}_\text{ek}\right)=1-\partial^{\mathrm\omega}\left(\text{G}^\ast\right)/\partial^{\mathrm\omega}\left(\text{G}^\ast\left({\text{v}}_\text{ek}\right)\right)$$

Where ${\text{I}\text{M}\text{C}}_{\text{G}}\left({\text{v}}_{\text{i}}\right)$ denotes the importance of node ${\text{v}}_{\text{i}}$ in supply chain network $\text{G}$. ${\text{I}\text{M}\text{C}}_{{\text{G}}^{\text{*}}}\left({\text{v}}_{\text{e}\text{k}}\right)$ denotes the importance of node ${\text{v}}_{\text{e}\text{k}}$ in supply chain node associative relationship (directed arc) network ${\text{G}}^{\text{*}}$. ${\text{M}}_{\text{e}\text{k}}^{\text{i}}$ is the set of nodes in ${\text{G}}^{\text{*}}$ corresponding to all directed arcs of ${\text{v}}_{\text{i}}$ in $\text{G}$. ${\text{q}}_{\text{i}}$ is the number of neighboring nodes with node ${\text{v}}_{\text{i}}$. $\sum _{\text{k}\in {\text{M}}_{\text{e}\text{k}}^{\text{i}}}{\text{I}\text{M}\text{C}}_{{\text{G}}^{\text{*}}}\left({\text{v}}_{\text{e}\text{k}}\right)/{\text{q}}_{\text{i}}$ is the sum of the importance of all association relations (directed arcs) generated by node ${\text{v}}_{\text{i}}$. $\text{I}\text{M}\text{C}\left({\text{v}}_{\text{i}}\right)$ is the important index of node ${\text{v}}_{\text{i}}$.

(xxii) Normalizing the node importance index using Eq. (12) because ${\text{I}\text{M}\text{C}}_{\text{G}}\left({\text{v}}_{\text{i}}\right)$ may be greater than 1. where ${\text{I}\text{M}\text{C}}_{\text{f}}\left({\text{v}}_{\text{i}}\right)$ denotes the node important index of ${\text{v}}_{\text{i}}$ after normalization; N is the number of nodes in the network G.

12 $${\text{IMC}}_\text{f}\left({\text{v}}_\text{i}\right)=\text{IMC}\left({\text{v}}_\text{i}\right)/\sum_{\text{i}=1}^\text{N}\text{IMC}\left({\text{v}}_\text{i}\right)$$

#### Quantification of the supply chain’s ability to recover from risk

Rajesh and Ravi (2015) stated that SCR* is the ability of the system to recover to its original or a better state when suffering risk. Therefore, we consider the ability of a supply chain to recover from a risk interference as another dimension of SCR.

In KISCs, participants are often deeply bound to each other, meaning that a problem at one node will quickly propagate to others. For example, in a gaming software supply chain, if a situation occurs in one of the underlying libraries, it can affect the functionality of many pieces of middleware at the same time. Therefore, we use the SIR risk propagation model to simulate the change in the state of all nodes in the KISC network after a risk interference. We measure the recoverability index of KISCs by calculating the time from the onset of suffering a risk until most nodes are out of risk.

The SIR model was initially used for the propagation of viruses, but there are some similarities between the propagation of risk in the SCN and the propagation of viruses. Firstly, in the propagation environment, both are propagated on complex networks. Secondly, in the propagation process and direction, both are propagated among entities with related relationships and are bidirectional. Finally, the propagated targets have different risk resistance. So, the propagation of risk in SCN can be done with the help of the SIR model (Kabir et al. 2020).

**Fig. 2:**
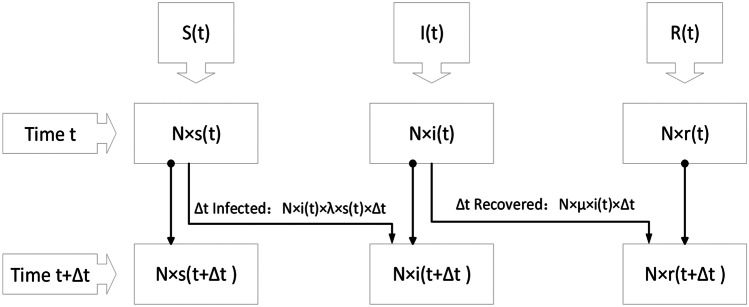
The state transfer process of the SIR model

Angstmann et al. (2017) indicated that in the SIR risk propagation model, firms in SCN have three states, including the state $\text{S}$ of being easy to be interfered with by risk, the state $\text{I}$ of having been interfered with by risk, and the state $\text{R}$ of having been immune to risk. The state of firms will shift among the three states when suffering risk, and the process is shown in Fig. 2. The differential equation for state transfer between the three states are (13), (14), and (15).

13 $$\frac{\text{d}\left(\text{s}\left(\text{t}\right)\right)}{\text{d}\left(\text{t}\right)}=\frac{\text{d}\left(\frac{\text{S}\left(\text{t}\right)}{\text{N}}\right)}{\text{d}\left(\text{t}\right)}=\frac1{\text{N}}\left(\frac{\text{d}\left(\text{S}\left(\text{t}\right)\right)}{\text{d}\left(\text{t}\right)}\right)=\frac1{\text{N}}\left(-\mathrm\lambda\text{I}\left(\text{t}\right)\frac{\text{S}\left(\text{t}\right)}{\text{N}}\right)=-\lambda \text{i}\left(\text{t}\right)\text{s}\left(\text{t}\right)$$

14 $$\begin{aligned}\frac{\text{d}\left(\text{i}\left(\text{t}\right)\right)}{\text{d}\left(\text{t}\right)}&=\frac{\text{d}\left(\frac{\text{I}\left(\text{t}\right)}{\text{N}}\right)}{\text{d}\left(\text{t}\right)}=\frac1{\text{N}}\left(\frac{\text{d}\left(\text{I}\left(\text{t}\right)\right)}{\text{d}\left(\text{t}\right)}\right)\\&=\frac1{\text{N}}\left(\lambda{\text{I}}\left(\text{t}\right)\frac{\text{S}\left(\text{t}\right)}{\text{N}}-\mu\text{I}\left(\text{t}\right)\right)=\lambda \text{i}\left(\text{t}\right)\text{s}\left(\text{t}\right)-\mu \text{i}\left(\text{t}\right)\end{aligned}$$

15 $$\frac{\text{d}\left(\text{r}\left(\text{t}\right)\right)}{\text{d}\left(\text{t}\right)}=\frac{\text{d}\left(\frac{\text{R}\left(\text{t}\right)}{\text{N}}\right)}{\text{d}\left(\text{t}\right)}=\frac1{\text{N}}\left(\upmu{{\text{I}}}\left(\text{t}\right)\right)=\upmu{\text{i}}\left(\text{t}\right)$$

Where N represents the total number of firms in the supply chain, in our network model, N equals the number of network nodes multiplied by 4. $\text{S}\left(\text{t}\right)$ is the number of firms that are not interfered by risk but are in state $\text{S}$ at moment $\text{t}$, $\text{s}\left(\text{t}\right)=\text{S}\left(\text{t}\right)/\text{N}$. $\text{I}\left(\text{t}\right)$ is the number of firms that have been interfered with by risk in state $\text{I}$ at moment $\text{t}$, $\text{i}\left(\text{t}\right)=\text{I}\left(\text{t}\right)/\text{N}$. $\text{R}\left(\text{t}\right)$ is the number of firms that have been immune to risk interference in state $\text{R}$ at moment $\text{t}$, $\text{r}\left(\text{t}\right)=\text{R}\left(\text{t}\right)/\text{N}$. λ denotes the risk propagation rate of firms that have been interfered with by risk. ${\upmu }$ denotes the recovery rate of firms already interfered with risk.

As seen from the state transfer equation of the SIR model, the most critical is the risk propagation rate λ and recovery rate µ of the supply chain participants. Treating SCN as a bidirectional risk propagation network satisfies the requirements of the SIR risk propagation model. Then, we perform a weighted average of the overall risk propagation rate λ and recovery rate µ of SCN based on the actual situation of each node.

In measuring the risk propagation rate, we use the concept of the OWA operator proposed by Ahn (2006), considering the probability of a risk event occurring and risk propagation range. From objective factors, generally, the higher the node importance, the more internal influencing factors of the node and the more likely the risk will occur, so the magnitude of risk occurrence probability of each node is quantified as the node importance index ${\text{IMC}}_{\text{f}}\left({\text{v}}_{\text{i}}\right)$. There is a positive correlation between the risk propagation range and the degree of association between nodes, so the value of the risk propagation range can be quantified as the weights of the directed arc between nodes. So, the uniform risk propagation rate within SCN is set as a function of the node importance and the weight of the directed arc in Eq. (16).

16 $$\lambda =\text{f}_{1}\left(\frac{\sum_{\text{i}=1}^{\text{N}}\text{IMC}_{\text{f}}\left(\text{v}_{\text{i}}\right)\times \frac{\sum_{\text{j}\in \text{q}}\omega_{\text{ij}}}{\text{q}}}{\text{N}}\right)(0 < \lambda < 1)$$

The node recovery rate is quantified using the method proposed by Zuo (2019). Considering the vulnerability index, the higher the vulnerability index, the more the supply chain is affected by the risk and the slower the recovery rate. Therefore, the uniform risk recovery rate within the SCN is set as a function of the inverse of the SCV in Eq. (17).

17 $$\mu ={\text{f}}_{2}\left(\frac{1}{\text{SCV}}\right)\left(0<{\upmu }<1\right)$$

It is worth noting that the equation here only needs to ensure that λ and µ is in the range (0,1). Moreover, we make the following two assumptions about the initial state of the KISC participants.

##### Assumption 1

At the initial moment when the risk interference is encountered, only a very small percentage of firms are in states and. Therefore, the initial value of are set to 0.95, 0.03, and 0.02, respectively.

##### Assumption 2

The time scale of the risk interference is much smaller than the survival cycle of the system, which means that there are no firms not recovered in the risk simulation.

**Fig. 3:**
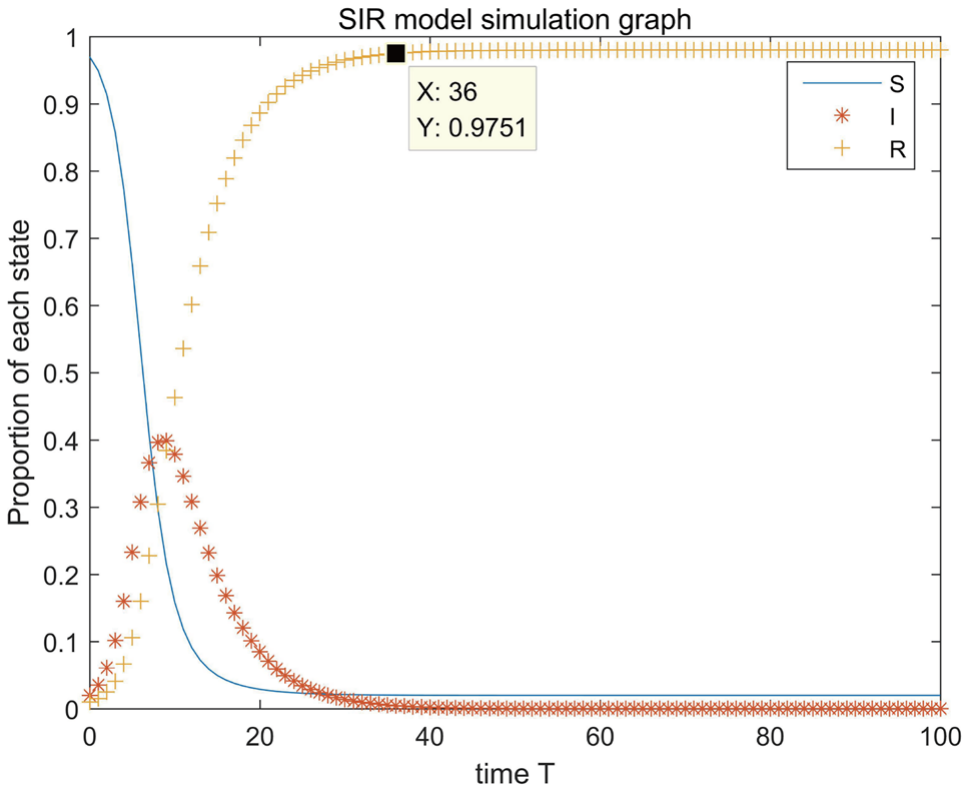
Explanation of recovery time T

Simulations are performed by Eqs. (14, 15, and 16). We can simulate the state transition process of the supply chain participants through Matlab, as shown in Fig. 3. We obtain the time $\text{T}$ from the beginning of risk interference to the time when most firms are out of risk. The ability of KISCs to recover from risk is quantified by the time T. As shown in Eq. (18).

18 $${\text{SCR}}^\ast=\frac{1}{{\text{T}}_{\text{r}\left(\text{t}\right)\approx 1}}$$

#### Calculation of supply chain resilience index

Based on the summary of SCR in the introduction and the literature review, we describe SCR as two dimensions, the ability of the supply chain to resist risk and recover after suffering risk. We assess these two ability indexes using Eqs. (8) and (18).

Some scholars have reviewed quantitative research on SCR, stating that SCR is a function of SCV and SCR* (Hosseini et al. 2019; van der Vegt et al. 2015). Moreover, Longo and Ören (2008) indicated a certain negative relationship between SCR and SCV. Birkie et al. (2017) also highlighted a significant positive relationship between SCR and SCR*. So, the calculation of the resilience index is shown in Eq. (19), where ${\upepsilon }$ denotes the adjustment coefficient, ensuring that the SCR index is positive.

19 $$\text{SCR}={{\upepsilon }\text{SCR}}^\ast/\text{SCV}$$

It is worth noting that the strength of this paper’s assessment of SCR is that a resilience index can describe it. Although we have yet to have an explicit range of resilience indexes to tell how good or bad resilience is, this is not the focus of our attention. Our focus is on the change trends in SCR and the improvement of SCR.

### Supply chain resilience improvement paths

Previous literature on resilience improvement is mainly based on changing the network topology (Pavlov et al. 2018), link prediction (Lopez and Ishizaka 2019), supplier selection game (Rajesh and Ravi 2015), and product safety stock (Vimal et al. 2022). However, changing the original network structure of KISCs, increasing the association among network nodes, theoretical supplier selection, and excessive product inventory will likely damage some firms’ original interests significantly. Therefore, we draw on the “structure-process-redundancy” value-added network (Ivanov and Dolgui 2019). We propose two ways of resilience improvement. One is to improve firms’ development capacity, and the other is the moderate industrial backup.

#### Improve firms’ development capacity

For the directed arc weights representing the associative relationships among firms. Some studies pointed out that SCV most likely originates from the associative relationships between supply chain participants (Ojha et al. 2018; Pournader et al. 2017). Moreover, the description of SCR* also depends heavily on the closeness of the associative relationships. So, SCR may be influenced by the directed arc weights. Simchi-Levi et al. (2018) found that process flexibility can be a potential risk mitigation tool. Uddin and Akhter (2022) considered the positive impact of collaboration on supply chain capability. In KISC-directed weighted networks, process flexibility and supply chain collaboration refer to the improvement of firms’ development capacity, expressed as a change in the weight of the directed arc.

According to the description of the directed arc, it represents the development capacity of a class of firms. We can analyze the meaning of the change in the directed arc weights numerically, ${\upomega }=\text{S}\text{G}\text{R}\times \text{P}\text{G}\text{R}/{\text{M}\text{C}\text{R}}_{4}$. If a node’s directed arc weight increases, the degree of associative relationships will become stronger. Because the rise of SGR or PGR means that the node has a better business condition and innovation level, and the decrease of ${\text{M}\text{C}\text{R}}_{4}$ implies that such firms’ market structure will be more stable. Therefore, theoretically, SCR will be improved regardless of whichever node’s directed arc weight is raised.

#### Industrial backup

From the perspective of network nodes, node redundancy brings some robustness (Bode and Wagner 2015), indicating that the more complex the network is, the lower its vulnerability will be. However, redundant nodes increase the possibility of risk in the supply chain and reduce recoverability. So, node redundancy may affect SCR (Yazdanparast et al. 2021), but this effect’s results are not clear. Ambulkar et al. (2015) argued that the continuous input and reallocation of resources must be considered in resilience improvement. Kumar et al. (2022) noted the need to consider reallocating supply chain resources or reinvesting resources. In this paper, the continuous input of resources is expressed as an industrial backup of a production process.

In our constructed directed weighted network, the industrial backup of a node is the addition of a new node with the same position as the original node in the SCN. The directed arc direction of the new node is the same as the backup node, and the weight is set to ${\uptheta }$ times the backup node. ${\uptheta }$ As the industrial backup intensity, ${{\upomega }}_{\text{n}\text{e}\text{w}}={\uptheta }\times {{\upomega }}_{\text{o}\text{l}\text{d}}$.

## Case analysis

The case analysis in this paper takes the global IC supply chain as an example. As a typical KISC, the IC supply chain has been most needed to manage resilience in recent years. The reason is that chips are the core component of all electronic devices today. The impact on the global electronics market is enormous when the IC supply chain is under risk interference. For example, under the influence of COVID-19, many car manufacturers have started to scale down production due to the lack of automotive-grade chips.

In addition, chip development is a long-term global goal. If there is no designated direction or no strategy for solid development, it may cause a tremendous waste of resources. Identifying node importance and assessing resilience in this paper provide directions for managing the IC supply chain. We provide theoretical support for creating a more resilient IC supply chain.

### Construction of a directed weighted network for the IC supply chain

**Fig. 4:**
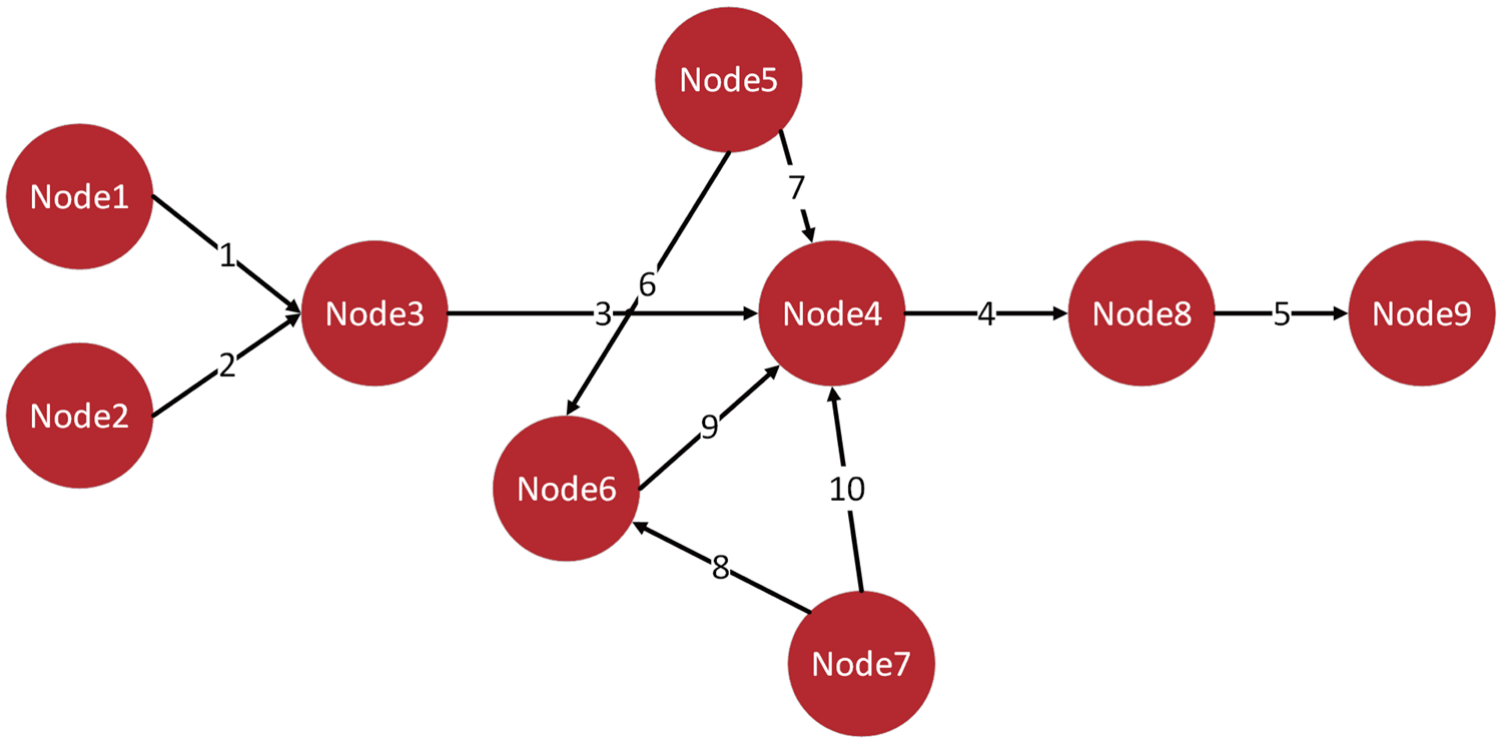
The IC supply chain directed weighted network

According to the construction method of the KISC networks, we select eight classes of firms in the IC industry as network nodes in Table 4. Based on the IC product production process, we establish a directed weighted network for the IC supply chain, as shown in Fig. 4. In quantitative indicator data collection, the $\text{S}\text{G}\text{R}$ data comes from the purchased SEMI industry reports, the $\text{P}\text{G}\text{R}$ data from the incoPat database, and the ${\text{M}\text{C}\text{R}}_{4}$ data from the China IC Industry Development Statistics Report. Then, according to Eq. (2), We quantify the weights of the directed arc from 2014 to 2020, as shown in Table 5.

**Table 4:** Description of network nodes in the IC supply chain

Node1	Electronic design automation (EDA) software firms
Node2	IP-based firms
Node3	fabless design firms
Node4	Integrated circuit manufacturing firms
Node5	Equipment manufacturing firms
Node6	Wafer manufacturing firms
Node7	Material production firms
Node8	Packaging and testing firms
Node9	The final product, not as an assessment node

**Table 5:** IC supply chain network data for 2014 to 2020

Edges/Year	2014	2015	2016	2017	2018	2019	2020
Edge1	3.38945	3.72186	3.47944	3.25184	3.40872	3.40289	3.07197
Edge2	3.49818	3.10002	4.38775	3.66156	3.55244	2.98054	3.30674
Edge3	2.94353	2.95952	3.05852	3.06535	3.00453	2.86228	2.71096
Edge4	3.36169	3.39349	3.12285	3.53289	3.36363	3.39198	3.36531
Edge5	4.20937	4.00270	4.00649	4.62014	4.49294	4.01727	4.11666
Edge6	3.74659	4.35901	3.57443	3.69574	3.94827	3.34186	3.56021
Edge7	3.53364	3.32414	3.82513	3.80873	3.54798	3.49528	3.20057
Edge8	3.42919	3.42062	3.16548	3.88023	3.64303	3.14364	3.32216
Edge9	4.06378	3.94029	4.27391	4.43432	3.59396	4.04523	4.09499
Edge10	3.54145	3.52201	3.50428	3.83630	3.65614	3.35423	3.37576

### Quantification analysis

**Fig. 5:**
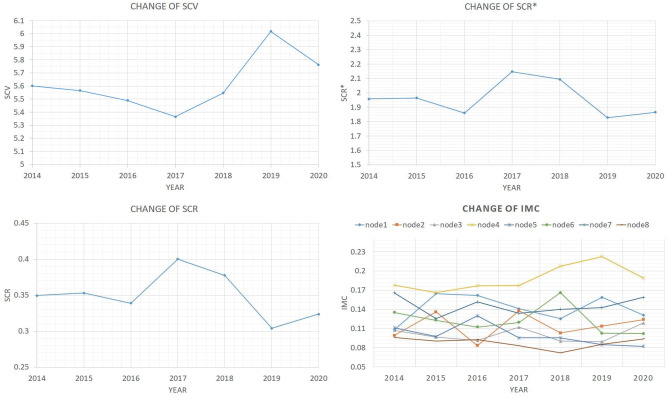
Quantification results of SCV, SCR*, node importance index, and SCR

Based on the collected data and the quantification method, the trends of SCV, SCR*, node importance index, and SCR of the global IC supply chain from 2014 to 2020 are obtained, as shown in Fig. 5. We should be more concerned with the change trends in the various indicators rather than specific values, as we do not have an exact value to indicate whether the situation is good or bad.

The quantified results show that.

(i) From the SCV and SCR* change trend graph, we find that before 2019, they generally remain stable and at a better level despite changes. In 2019, SCV rose from an average value of 5.5 to 6.0, a increase of 10%. SCR* also fell from a best case of 2.15 to 1.84, a reduction of 16.8%. This unpromising situation in 2019 continued until 2020.
  Based on our description of SCV and SCR*, this result illustrates the decreasing ability of the global IC supply chain to resist risk and recover after suffering risk. After analysis, this change is most likely related to global IC development environment changes. 2019 saw a global trade war between China and the U.S., followed by the worldwide outbreak of COVID-19 in 2020, which significantly impacted the IC supply chain. Suggesting that political factors and unforeseen events affect KISC to a large extent.
(ii) From the graph of node importance, we can find that IC manufacturing firms (node 4) have the highest node importance index, followed by EDA software firms (node 1) and material production firms (node 7). The node importance of packaging and testing firms (node 8) is always low.
  This result corresponds to the analysis of KISC characteristics. For KISCs, the key nodes often lie in the production processes with core technology patents or market monopolies. For example, IC manufacturing firms are the most critical nodes because ASML almost monopolizes high-precision lithography. EDA software firms are also in key positions in the supply chain due to their deep binding relationships and core technology patents on the IC ecosystem. Because of the relatively simple technical requirements of the packaging and testing firms, they are less important in the IC supply chain, despite being the necessary path for chip production.
  As the data shows consistently, China’s IC industry was severely damaged by the 2019 U.S.-China trade war precisely because the U.S. government sanctioned critical nodes in the IC supply chain. For example, the U.S. government restricted access to EDA software and forced TSMC, an IC manufacturer, not to provide services for Chinese IC design firms.
(iii) The trend graph of SCR shows that the resilience index in 2019 is at its lowest point in recent years, with a reduction of almost 33.4%. 2020 is still the same. The results of the resilience assessment are very closely aligned with the current worldwide demand for IC supply chain development because many countries have realized to create a more resilient IC supply chain.
  On June 8, 2021, the U.S. White House conducted a comprehensive supply chain assessment under Executive Order 14,017. It emphasized improving the resilience of the IC supply chain by rebuilding production and strengthening industry cooperation. The Chinese government has formulated relevant policies as early as 2020 in response to the reduced resilience of the IC supply chain, including tax reduction, increased investment, and enhanced international cooperation for the IC industry. The EU also officially announced the Chip Bill on February 8, 2022, which intends to use more than 43 billion euros to expand the IC supply chain.

In summary, based on the KISC-directed weighted network model, the resilience assessment of the IC supply chain aligns with the industrial reality. From a real-world perspective, nodes with high importance index in the KISC network should receive more attention. Therefore, in the next section, we will discuss the impact of two resilience improvement paths on different importance nodes.

### Simulation of supply chain resilience improvement paths

In this section, we use Matlab to investigate the effectiveness of the two resilience improvement paths through simulation. Moreover, the essential in the simulation is the benchmarking data. Therefore, we use the global IC supply chain data in 2020 as a comparison. Explicitly, the comparison data before resilience improvement are SCV = 5.7632, SCR*=1.6739, and SCR = 0.2904.

#### Simulation of improving firms’ development capacity

**Fig. 6:**
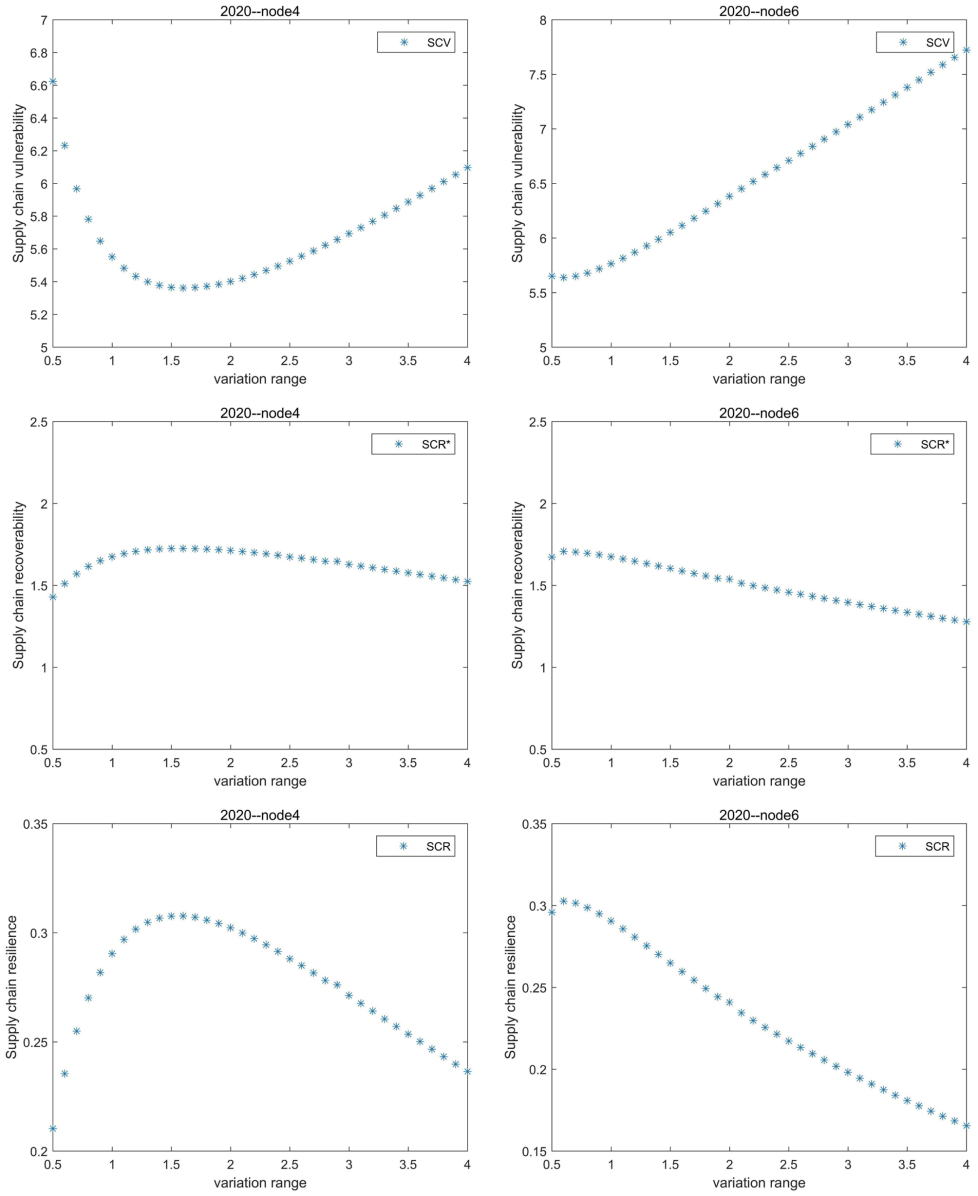
Changing trends of SCV, SCR* and SCR when improving firms’ development capacity of node 4 or node 6

According to the description of the directed arc weight in Section 4.1.3, it represents the development capacity of a class of firms. So, we vary the directed arc weights to study the changes in SCR. Combining with the node importance identified for the global IC supply chain in 2020, node 4 with the highest importance and node 6 with lower importance are distinguished to simulate separately as **(a) (b)**. The quantification results are shown in Fig. 6, where the horizontal axis is the multiplier of the change of the directed arc weight ${\upomega }$. $\text{x}=1$ indicates the initial state. The vertical axis represents the changing trend of SCV, SCR*, and SCR, respectively.

- For the IC supply chain in 2020, we improve the development capacity of such firms represented by node 4, the results are shown on the left side of Fig. 6.
- For the IC supply chain in 2020, we improve the development capacity of such firms represented by node 6, the results are shown on the right side of Fig. 6.

Firstly, the change in SCR is mainly due to the change in SCV because the SCR* varies very little. The change in SCV for node 4 reaches its lowest point when the weights of the directed arc change 1.5 times, a 15.3% reduction. Node 6 has a linear increase in SCV. Secondly, by increasing the development capacity of such firms represented by node 4, SCR is not continually improving but shows an inverted U-shaped. The highest level of the resilience index has a value of around 0.31. Finally, increasing the development capacity of such firms represented by node 6 will decrease SCR.

Realistically, for node 4, with high importance in the IC supply chain. On the one hand, a certain degree of improvement in such firms’ development capacity means that the market structure will be more solid, the business condition will be better, or the innovation level will be higher. Therefore, resisting risk is better, making a higher SCR. On the other hand, if firms’ development capacity is improved beyond a specific range, the deep binding relationship among participants can lead to the capability of other firms not matching node 4. For example, IC manufacturing firms meet the 3 nm production process, but the equipment manufacturing firms cannot provide a matching etcher. So, this situation will reduce SCR from a whole supply chain perspective.

**Fig. 7:**
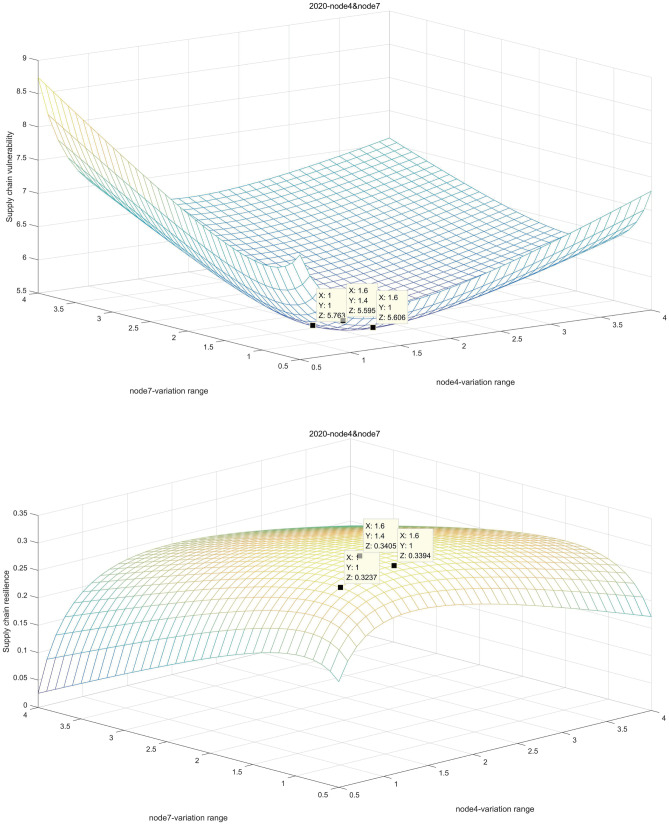
Changing trends of SCV and SCR when improving firms’ development capacity of node 4 and node 7

For node 6, the development capacity of such firms has met the technology or market demand in the IC supply chain. For example, China’s IC design and packaging firms have advanced globally. However, if there is a problem in the manufacturing firms, then there is no use for advanced design and packaging technologies, resulting in a low SCR of China’s IC supply chain.

(iii) The results in (a) and (b) show that improving firms’ development capacity is effective only for nodes with high node importance, mainly by affecting SCV and thus SCR. Therefore, node 4 and node 7, with high node importance in the IC supply chain, are targeted simultaneously. The results are shown in Fig. 7. The X-axis represents the changing range of the directed arc weights of node 4; the Y-axis represents the changing range of the directed arc weights of node 7; and the Z-axis represents the changing trend of SCV and SCR, respectively.

Through simulation, it can be found that the overall SCR shows an upward convex trend, indicating that simultaneously changing the directed arc weights of node 4 and node 7 can get a higher SCR. As illustrated in the figure, When the directed arc weights of node 4 and node 7 are expanded by a factor of 1.6 and 1.4, respectively, the value of the resilience index reaches 0.3405, which is about 10% higher than only changing the directed arc weight of node 4. In a realistic sense, simultaneously improving development capacity at multiple high-importance nodes can achieve a more efficient increase in SCR, suggesting that attention should be paid to a balanced development between the various classes of firms when improving SCR.

In summary, based on the simulation results in **(a) (b) (c)**, the following conclusions are drawn.

1. Improving firms’ development capacity affects SCR mainly through SCV, explaining that this way can increase the ability of the supply chain to resist risk. Moreover, significant improvement in SCR can only be achieved through high-importance nodes.
2. Due to the deep binding relationship among KISC participants, improving firms’ development capacity needs to be within a specific range to improve SCR effectively.
3. In practice, we can consider improving the development capacity of multiple nodes simultaneously to achieve a better significant SCR improvement.

#### Simulation of industrial backup

**Fig. 8:**
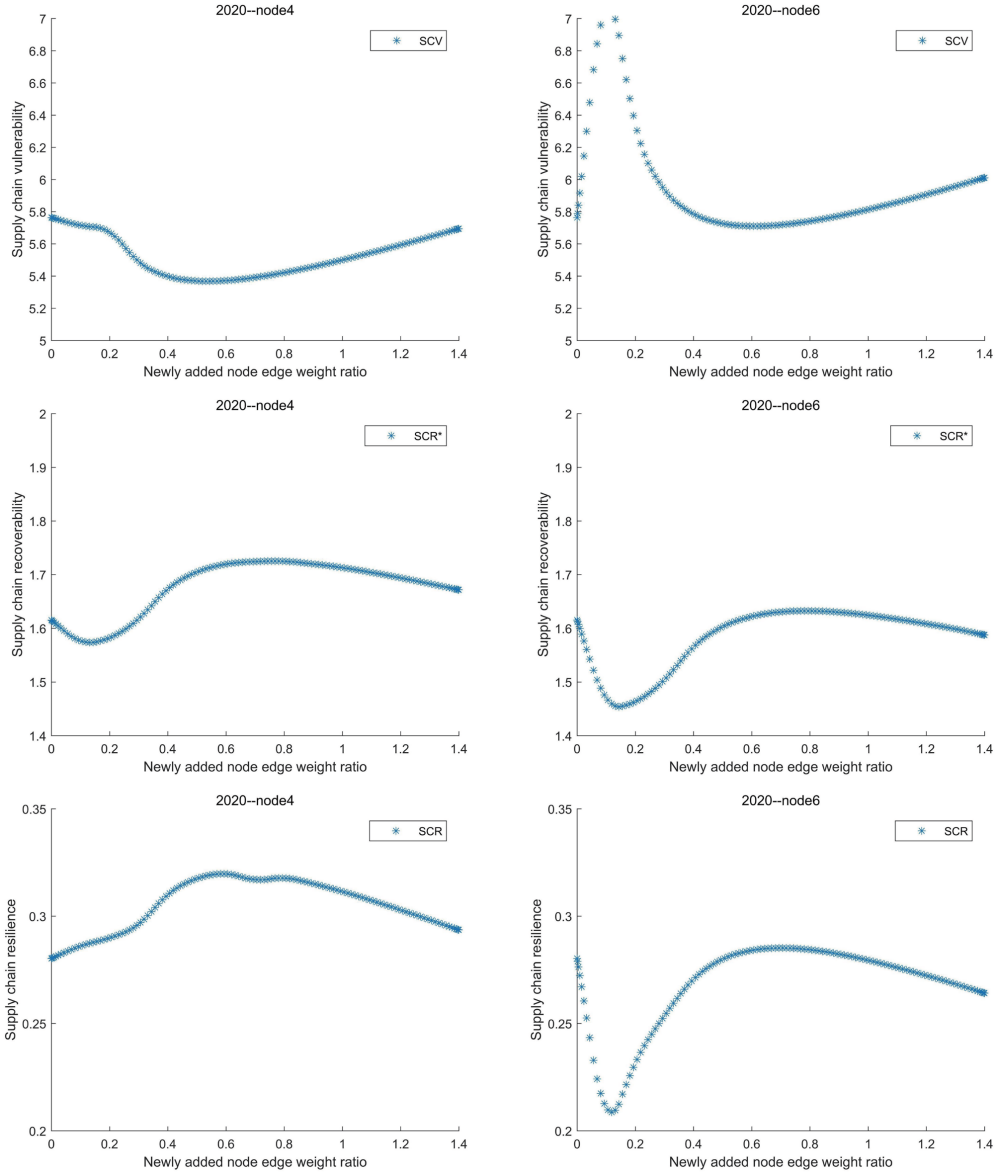
Changing trends of SCV, SCR* and SCR when industrial backup for node 4 or node 6

According to the analysis in Section 4.3.2, node redundancy is likely to affect SCR. Therefore, this section uses the concept of node redundancy. Industrial backup for nodes with different importance, which means adding new nodes. The directed arc direction of the new node is the same as the backup node, and the weight is set to ${\uptheta }$ times the backup node. ${\uptheta }$ as the industrial backup intensity, ${{\upomega }}_{\text{n}\text{e}\text{w}}={\uptheta }\times {{\upomega }}_{\text{o}\text{l}\text{d}}$. Industrial backup **(d) (e)** for node 4 with high importance and node 6 with low importance, respectively. The results are shown in Fig. 8. Where the horizontal axis represents the value of industry backup intensity ${\uptheta }$, $\text{x}=0$ is the state without node backup, and the vertical axis represents the trend of SCV, SCR*, and SCR.

(iv) The industrial backup for node 4. The results are shown on the left side of Fig. 8.
(v) The industrial backup for node 6. The results are shown on the right side of Fig. 8.

Firstly, the industrial backup is through SCV and SCR*, thus affecting SCR. The maximum range of variation in SCV and SCR* exceeds 10%. Secondly, for node 4, with high importance, the maximum fluctuation range of SCR is more than 15%. Thirdly, for the less important node 6, industrial backup has little impact on the SCR, and when ${\uptheta }$ is small, SCR appears very low.

Realistically, for node 4, with high importance in the IC supply chain, the industrial backup will ensure the firms’ substitutability in the market and technology. This substitutability will alleviate the technology or market monopoly, improving SCR. The industrial backup strength ${\uptheta }$ also needs to be within a specific range. Because when ${\uptheta }$ is relatively large, intense market or technology competition is triggered within such firms, then reducing the SCR.

For the less important node 6, the SCR does not increase because the market pattern and technical requirements of such firms have already met the current demand. When the industrial backup intensity ${\uptheta }$ is low, the stable market pattern is disrupted due to the sudden industrial backup, leading to a significant decrease in SCR. That is why the Chinese government implemented industrial backup for IC manufacturing firms but did not focus on the packaging and testing firms.

**Fig. 9:**
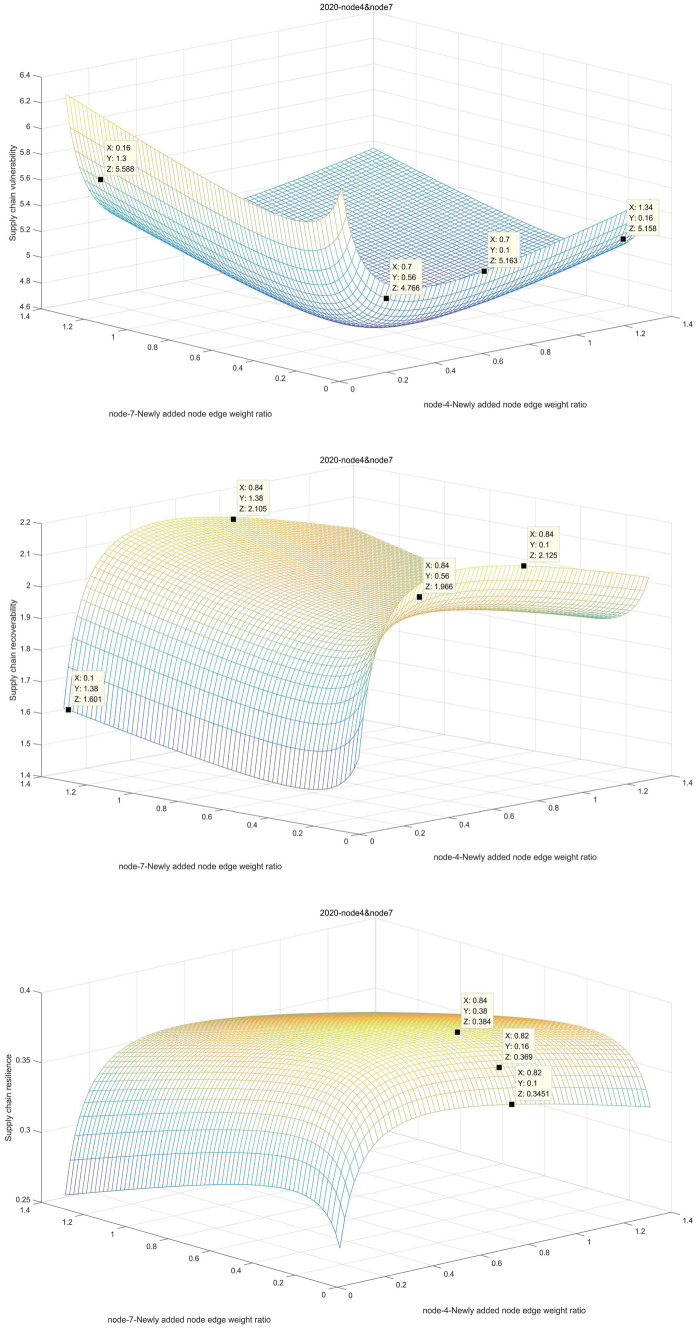
Changing trends of SCV, SCR*, and SCR when industrial backup for node 4 and node 7

(f) The results of **(d) (e)** show that the industrial backup needs to target the nodes with high importance and impact both SCV and SCR*. Therefore, the industrial backup is performed simultaneously for node 4 and node 7. The results are shown in Fig. 9. The X and Y axes indicate the variation range of the industrial backup intensity ${\uptheta }$ at node 4 and node 7, respectively. The Z-axis indicates the changing trend of SCV, SCR* or SCR, respectively.

As can be seen from the points marked in the SCV graph, a significant reduction in SCV only occurs when the two nodes have comparable level of backup and the value of ${\uptheta }$ is between (0.2, 0.6). The graph of SCR* shows that the intensity of industrial backup for node 7 is not critical, and only a higher level of backup for node 4 can effectively improve the SCR*. For SCR, when node 4 has a backup level ${\uptheta }$ of 0.84 and node 7 has a level ${\uptheta }$ of 0.38, the resilience index reaches a value of 0.384, a 32% increase compared to no measures.

In the real world, when the industrial backup is carried out simultaneously for multiple classes of firms, it will result in a more balanced technology backup or market distribution among supply chain participants, which is more valuable than a technological breakthrough at a single node.

In summary, the following conclusions are drawn for the industrial backup.

1. The industrial backup is through SCV and SCR*, thus affecting the SCR. This means that industrial backup not only increases the ability of the supply chain to resist risk but also improves its ability to recover.
2. The industrial backup intensity must be within a specific range and cannot cause market competition among firms.
3. When resources allow, industrial backup can be considered for multiple important nodes in the supply chain.

## Conclusion and outlook

### Conclusion

In this paper, by analyzing the product production process in KISCs, we identify two characteristics that distinguish them from non-KISCs, including the high degree of modularity in product production and the deep binding relationships among participants. Therefore, we develop a directed weighted network model more suitable for KISCs. This network structure clearly describes the location and role of KISC participants and indicates their relationships. In the resilience assessment, we express SCR as a comprehensive capability, including the ability to resist risk and recover from it. We quantify these two ability indexes and identify the node importance index. More importantly, we have proposed two resilience improvement paths for KISCs. The case analysis leads to the following conclusions.

(i) Expressing SCR as a comprehensive capability, the resilience assessment based on the directed weighted network for KISCs is in line with the actual situation of the industry. For example, the resilience assessment of the IC supply chain shows a significant decrease in 2019 and 2020. The considerable reduction in SCR is due to the U.S.-China trade war in 2019 and the outbreak of COVID-19 in 2020.
(ii) The two proposed resilience improvement paths are effective. For the IC supply chain, many countries have realized the importance of improving the development capacity of firms and industrial backup. For example, the Chinese government had invested heavily in industrial backup for the IC supply chain, and the U.S. government, through its 2021 Supply Chain Assessment Report, had begun to focus on the development capacity of firms in the IC supply chain.
(iii) The management of resilience needs to focus on the key nodes in KISCs. Moreover, the degree of improvement for firms’ development capacity and the intensity of industrial backup must be within a specific range. In KISCs, highly important nodes often have market monopolies or core technology patents. Improving the development capacity of these key nodes or conducting industrial backup can alleviate the market monopoly and technology gap, creating a degree of substitutability. However, if the degree of firms’ development capacity improvement or the intensity of industrial backup is too high, it will likely result in a technology mismatch or fierce competition among firms.

### Theoretical contribution and management implications

Theoretically, this study offers a new way to build supply chain networks, especially for supply chains with deep associative relationships among participants. A class of firms can be treated as network nodes, and quantitative indicators affecting the relationships among nodes are used as weights for the directed arc. In resilience assessment, a more holistic approach is proposed, which needs to consider the supply chain’s comprehensive capability to resist risk and recover from it. Importantly, two resilience improvement paths are provided for supply chains where technology, knowledge, and experience are the main factors of production.

In the managerial sense of this paper, our findings can support the decisions of two types of managers.

The first category is for managers of firms in KISCs. On the one hand, the formulation of firm development strategies should be in line with the characteristics of the supply chain. For example, it is more critical for the chip supply chain to maintain technology matching and market equilibrium rather than product inventory and logistics levels. On the other hand, constructing a firm’s technology system should align with the technological requirements of upstream and downstream firms, i.e., the firms need to identify their position in the SCN. For example, in the software supply chain, the choice of programming language should incorporate the upstream base library’s development environment and meet the downstream products’ performance requirements.

The second category is for KISCs’ industrial policy managers. From the analysis of the examples in this paper, improving the resilience of KISCs requires a balanced development among firms. Therefore, the following points must be focused on in policy formulation for managing SCR. Firstly, policy objectives should not be centered on suppressing competitors, which must maintain a technological balance among firms. Secondly, innovation-based policies need to be highlighted to consolidate existing technological advantages. Finally, there is a need to maintain a balanced market by continuously supporting small and medium-sized enterprises.

### Limitations and future research

The results of this study should be interpreted while considering its limitations. When constructing KISC-directed weighted networks, we do not consider the phenomenon of technology stratification in KISC networks. For example, the manufacturing process may be 7 nm, 14 nm, or 28 nm in the chip supply chain. If the 7 nm process cannot meet the current production demand, investing resources in a 28 nm line makes no sense. Therefore, although the results of this study are highly relevant, there should be caution in the specific measures of resilience improvement. The two resilience improvement paths we propose will be more targeted if the technology stratification is carried out in a subsequent study.

In our assessment of SCR, we have selected three quantitative indicators related to the characteristics of KISCs. However, this does not exclude the uncertain influence of other factors. So, future studies can consider more factors to achieve a more comprehensive assessment of SCR. For example, we can incorporate cost and risk factors to achieve lower costs and a less risky resilience improvement.

Our research is set in the context of KISCs, so the methods in this paper cannot be directly applied to other supply chains. However, it is easy to use the network model we have constructed to assess SCR as long as the relevant influencing factors are found according to the supply chain characteristics and the quantification method of the directed arc weights is changed.

## Data Availability

The relevant data for this paper can be obtained in the SEMI(https://www.semi.org/en) and IncoPat(https://www.incopat.com/).
